# Characterizing the Structural Conformation of Highly Charged Star-Linear Polyelectrolyte Mixtures in Solution

**DOI:** 10.1021/acs.macromol.5c01180

**Published:** 2025-09-16

**Authors:** Utku Gürel, Ilija A. Gjerapic, Wouter J. H. Arends, Roshan Akdar Mohamed Yunus, Aleksander Guzik, Patrizio Raffa, Daniele Parisi, Andrea Giuntoli

**Affiliations:** † Zernike Institute for Advanced Materials, 3647 University of Groningen, Nijenborgh 3, 9747AG Groningen, The Netherlands; ‡ Department of Chemical Engineering, Engineering and Technology Institute Groningen, 332143 University of Groningen, Nijenborgh 3, 9747AG Groningen, The Netherlands

## Abstract

Long-range electrostatic interactions provide unique opportunities to tune the conformation and phase behavior of polymeric micelles and soft colloids in solution, but their effects remain understudied due to the higher synthesis, characterization, and simulation complexity. We recently showed that micelles with long, charged polymer arms exhibit unique softness and glassy behavior at varying concentrations due to long-range electrostatic interactions, and developed a molecular dynamics model to validate the experimental results. Here we further explore our new system, and we investigate mixtures of highly charged star polyelectrolytes (SPEs, mimicking spherical micelles) and oppositely charged linear polyelectrolytes (LPEs) using molecular dynamics simulations and rheological validation. SPE size and conformation are strongly affected by LPE addition, which introduces charge neutralization within the SPEs’ bounding spheres, leading to shrinkage or expansion depending on the LPE length and concentration. Long LPEs form bridges between multiple SPEs, inducing clustering and promoting liquid–liquid phase separation at high charge ratios, triggering a glass-to-coacervate transition. Experimental rheology confirms that increasing the LPE initially decreases, then drastically increases viscosity, together with visual phase separation of the system, validating the simulation results. These findings highlight the interplay between electrostatic interactions, chain entropy, and packing effects, offering insights into how polyelectrolyte mixtures can be tuned for controlled complexation and phase behavior in soft materials design.

## Graphical abstract

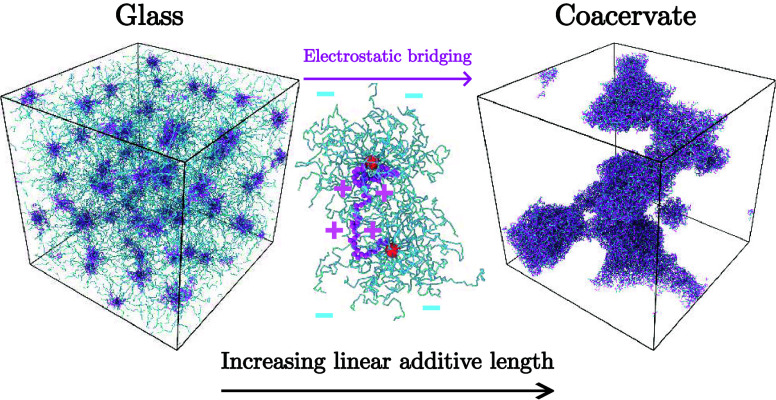

## Introduction

Star polyelectrolytes (SPEs), branched polymers of multiple charge-bearing linear chains grafted to a common core, provide an ideal model system to study fundamental soft matter.^1^ The well-defined star architecture, characterized by a precise arm number and length, offers high control over interstar interactions and generally serves as a good model for more complex structures, such as micellar systems^2,3^ and polymer-grafted nanoparticles.^4–6^ This design places them between linear polymer chains and hard colloidal particles in terms of softness, making them transitional forms whose softness can be tuned by varying the molecular architecture, chain flexibility, and distribution of charged units.^7,8^ The dense polymeric shell around the star’s core and the charge density along the arms allow systematic tuning of steric and electrostatic effects, respectively, thereby creating mesoscopic structures with unique properties.^9^

In addition to their fundamental significance, star polyelectrolytes have shown promise in ion conduction for enhanced battery performance,^10–12^ in multiresponsive microcapsules for controlled loading and release,^13^ and in protein-based processes requiring high binding capacities.^14,15^ However, limited understanding of their complex structure–property relationships leads to persistent bottlenecks, including the challenge of balancing mechanical robustness with ionic conductivity,^11^ maintaining stable permeability transitions in microcapsules across broader operating conditions,^13^ and preventing unwanted phase separation or protein denaturation in biochemical applications.^15^

Over the past three decades, significant theoretical advancements have been made in understanding the concentration-dependent behavior and complex electrostatic interactions of star polyelectrolytes.^1,9,16–31^ Early studies, such as those by Borisov and Zhulina, developed scaling theories for strongly charged star polyelectrolytes in dilute solutions, demonstrating that their size is less sensitive to ionic strength than their linear counterparts.^18^ Building on this, Wolterink et al. utilized numerical self-consistent field approaches and analytical theories to show that counterions localized within the intrastar space effectively screen intramolecular electrostatic repulsion in many-armed stars, with the degree of screening decreasing as the number of arms is reduced.^19^ Expanding on this work, Jusufi et al. investigated the conformational properties of star polyelectrolytes using molecular dynamics simulations combined with analytical free-energy calculations. They developed an effective potential between two star polyelectrolytes, revealing that entropic contributions from trapped counterions dominate the effective interaction at high overlaps, while electrostatic effects diminish due to near-complete neutralization.^20,21^ Similarly, Shusharina and Rubinstein focused on structural differences in star polyelectrolyte solutions, introducing a scaling theory that accounts for the presence or absence of condensed counterions across different concentration regimes.^26^ Researchers have also focused on interactions between star polyelectrolytes and oppositely charged components. Likos et al. explored the formation of novel complexes involving charged colloidal particles, providing insights into the interplay between electrostatic interactions and particle architecture.^9^ More recently, Chremos et al. investigated how polyelectrolyte topology, including stars, affects counterion binding and clustering.^29^ They further showed that moderately branched star polyelectrolytes exhibit particle-like properties and form an amorphous solid at high polymer concentrations.^30^ Larin et al. employed molecular dynamics simulations to investigate complexes formed by charged stars and oppositely charged linear polyelectrolytes, predicting a critical charge ratio of 0.5, beyond which phase separation occurs.^32^ Similarly, Ni et al. studied the behavior of complexes formed by spherical polyelectrolyte brushes and oppositely charged linear polyelectrolytes, identifying a swelling-collapse-reswelling mechanism driven by variations in linear chain concentration.^33^ Further studies have examined complexes involving diblock copolymer micelles. Kalogirou et al. demonstrated that the size of complexes formed by diblock copolymer micelles and oppositely charged linear polyelectrolytes varies nonmonotonically with the charge ratio, showcasing the intricate balance of electrostatic and structural effects in these systems.^34^

These investigations have significantly advanced our understanding of isolated star polyelectrolytes and their assemblies with oppositely charged linear chains. They reveal how the distinct branching and charge distribution of SPEs can induce emergent behaviors, ranging from near-complete neutralization at high overlaps to swelling–collapse transitions, by tuning parameters such as the degree of branching, ionic strength, and concentration. However, these studies have largely focused on single-star systems, leaving a gap in understanding how a collection of star polyelectrolytes interact with both oppositely charged species and each other, especially in the high concentration regime relevant for biological processes and material design.

We build on our previous work,^35^ where we modeled charged spherical micelles derived from self-assembled amphiphilic block polyelectrolyte^36^ and demonstrated their glass-forming behavior. This study extends the investigation by conducting a systematic theoretical analysis of a similar but scaled-down molecular dynamics model with the addition of oppositely charged linear polyelectrolyte additives using coarse-grained molecular dynamics simulations, and validates the theoretical findings with rheology. Thanks to the qualitative universal behavior, the scaled-down model allows us to perform this study in a much larger design space, while the previously used larger experimental systems provide qualitative proof of our main theoretical findings. The earlier study shed light on the glass transition behavior of soft-charged colloids. It demonstrated the potential of polyelectrolytes as a tool for tuning the rheological properties of colloidal systems.^35^ The primary goal of this work is to investigate the competing effects of particle packing, controlled by varying the concentration of star polyelectrolytes, and the entropic effects of oppositely charged species, which are modulated by altering their connectivity: from single counterions to linear polyelectrolytes of varying lengths and charge ratios. Understanding the interplay between electrostatic interactions and chain conformational entropy is essential for predicting how polyelectrolyte complexes assemble, adapt to changing conditions, and exhibit emergent properties such as phase separation and structural reentrance. These systems operate across multiple length scales, from the molecular dimensions of each charged segment to the overall size of the star, creating a rich phase space where variations in packing density and ionic strength drive significant changes in mesoscopic structure.

In this work, we show that star–star bridging and charge neutralization drive the structural and phase behavior of SPE-LPE mixtures. These effects culminate in local clustering, structural reentrance, and eventually phase separation at high charge ratios with sufficiently long LPEs. By combining coarse-grained molecular dynamics (CGMD) simulations with rheology, we characterize the rich behavior of these mixtures in the high-concentration regime. We demonstrate how chain length, concentration, and charge ratio collectively tune conformation and phase behavior. These findings set the stage for systematic future studies on liquid–liquid phase separation, macromolecular dynamics, counterion effects, and partial ionization, ultimately guiding the design of advanced soft materials.

## Methods

### Model

The structural properties of star polyelectrolyte (SPE) and linear polyelectrolyte (LPE) mixtures in solution are investigated using CGMD simulations. The self-assembled micelles in solution are modeled by a single neutral bead representing the hydrophobic core, with *f* polyelectrolyte arms grafted on it. Each SPE arm is composed of *M* beads. The beads represent one Kuhn segment of a polymer chain as employed in the bead–spring model.^37^ Nonbonded interactions between two beads are described by the shifted Lennard-Jones (LJ) potential

1 $$U_{\mathrm{LJ}}\left(r\right)=4\varepsilon \left[{\left(\frac{\sigma }{r-\Delta }\right)}^{12}-{\left(\frac{\sigma }{r-\Delta }\right)}^{6}\right],r<r_{c}+\Delta$$

where ε is the interaction strength, σ is the diameter of a monomer within a polyelectrolyte arm, Δ is the interaction shift, *r* is the distance between two beads, and *r*_c_ is the unshifted interaction cutoff. The shift value is set to 1.0 between two SPE cores and to 0.5 between the SPE core and the remaining particle types. Energy (ε), mass (*m*), and length (σ) scales are set to unity, leading to a unity simulation time (τ) defined as $\tau =\sqrt{m\sigma^{2}/\varepsilon }$ . The Boltzmann constant is also set *k*_B_ = 1 for convenience. The neutral core is assigned a diameter of 2σ and a mass of 8*m*, maintaining the mass density equal to that of the arm beads. Bonded interactions are modeled with a stiff harmonic potential

2 $$U_{H}\left(r\right)=k{\left(r-r_{0}\right)}^{2}$$

where *k* = 2500 ε/σ^2^, *r*_0_ = 1σ for monomer–monomer bonds, and *r*_0_ = 1.5σ for core-monomer bonds.^30^ The SPE arms carry a unit charge of −*e*, with the presence of either counterions, LPEs, or both, each having beads with charge +*e* to ensure the charge neutrality of our simulations. The long-range Coulomb interactions are given by

3 $$U_{\mathrm{Coulomb}}\left(r\right)=k_{B}T\cdot l_{B}\cdot \frac{z_{i}z_{j}}{r}$$

where

4 $$l_{B}=\frac{e^{2}}{4\pi \varepsilon_{0}\varepsilon_{r}k_{B}T}$$

is the Bjerrum length, which is the length scale at which the electrostatic interactions become comparable to random fluctuations, *e* is the elementary charge, *z*_*i*_ and *z*_*j*_ are the dimensionless valency numbers of particles *i* and *j*, ε_0_ is the vacuum permittivity, and ε_*r*_ is the relative dielectric constant. A cutoff of *r*_c_ = 10σ is used for Coulomb interactions.^30^ The Bjerrum length is set to 1 for all simulation stages involving charges.^38^ The long-range Coulomb forces are calculated using the particle–particle-particle-mesh (PPPM) method.^39^

### Simulation Parameters

To investigate the effects of microscopic changes on macroscopic properties, a solution of *N* = 64 individual SPEs is simulated at varying polymer volume percentages *c* in a periodic cubic box. The SPE architecture is defined as *f* = 32, M = 40 where *f* is the number of arms and *M* is the arm length, following the star polymer architecture convention. Solution concentration is defined as the Kuhn segment volume density of SPEs and ranges from *c* = {0.01, 0.02, 0.05, 0.1, 0.2, 0.5, 1.0, 2.0, 5.0, 10.0%}. LPEs are introduced at varying ratios and lengths to explore condensation effects. Pure SPE solutions contain *N f M* positively charged monovalent counterions. For star-linear PE mixtures, we define the charge ratio β as

5 $$\beta =\frac{LN_{\mathrm{LP}}}{\mathrm{NfM}}$$

where *L* is the number of beads in an LPE, ranging from {0.1M, 0.5M, M, 2M} for each β, and *N*_LP_ is the number of LPEs.^33^ β represents the total LPE charge relative to the total SPE charge. We consider β values in the range {0.0, 0.25, 0.5, 0.75, 1.0} where β = 0 corresponds to pure SPE solutions without LPEs, β = 0.5 an equal mixture of monovalent counterions and LPE beads, and β = 1.0 a pure mixture of SPEs and LPEs without counterions. We focus on the range 0 ≤ β ≤ 1.0 to consider SPE solutions only and treat the LPEs as additives. Four statistically independent runs with different initial particle positions and velocities are simulated for each point in the data space. The measured quantities are averaged over these runs, their means are reported, and the error bars are the standard error of the mean.

### Simulation Details

Before running simulations, particles are randomly packed in a sufficiently large simulation box and assigned random velocities sampled from a Maxwell–Boltzmann distribution corresponding to a temperature of *T* = 1.0. The integration time step is set to d*t* = 0.005τ. A soft relaxation step is run at a low concentration for 50τ using the attractive part of the LJ potential for core–core interactions (*r*_c_ = 2.5) and purely repulsive interactions for the remaining particles (*r*_c_ = 2^1/6^), ensuring no bead overlaps. The system is then compressed to the target concentration over 500τ. Equilibration at this concentration is performed for 5 × 10^3^τ using an NVT thermostat. Subsequently, a Langevin thermostat and NVE ensemble are used for further equilibration over 10^5^τ. The entire system remains neutral up to this point. Neutral star arms are gradually charged to −1; counterions and LPEs are charged to +1 for 500τ. A smooth, linear ramp of all particle charges is applied for 500τ to avoid instantaneous changes in molecular interactions. This time interval is long enough compared to the diffusive time of the counterions. After the ramp, the charges are fixed, and the simulation is further equilibrated for another 500τ, so that ion distributions and local arm conformations can reach equilibrium. Finally, the fully charged system is equilibrated for an additional 5 × 10^3^τ. Single-molecule properties are stable over this time and are averaged over different configurations. Collective properties are measured at the end of the equilibration. Simulations are carried out with the Large-scale Atomic/Molecular Massively Parallel Simulator (LAMMPS) software with periodic boundary conditions in all three dimensions.(https://www.lammps.org/).^40^ Snapshots are rendered with OVITO.^41^

### Experimental Details

Micelles were formed through the self-assembly of 4-arm polyelectrolyte stars in water, without the addition of salt. These stars consist of a polystyrene (PS) core and an outer polymethyl acrylic acid (PMAA) block. The molar masses of styrene and methacrylic acid monomeric units are 104 and 86 g/mol, respectively, with each arm containing 23 and 367 monomers. Further details on the synthesis and characterization can be found in previous studies.^36^ Note that the PS core remains inert after micellar self-assembly, and in the simulations of this work each star polyelectrolyte (SPE) corresponds to an entire micelle with a neutral core.^35^

To investigate the interaction between linear polyelectrolytes and charged micelles, the polymer *N*-[3-(Dimethylamino)­propyl]­methacrylamide (PDMAPMA) was added to a multiarmed PS–PMAA polyelectrolyte solution. For the synthesis of the linear polyelectrolyte, 4.95 g of DMAPMA (Sigma-Aldrich, ≥99%, free base monomer) was dissolved in 9.3 mL of DMSO without prior removal of the inhibitor. To this solution, 12.6 mg of 2,2′-azobis­(2-methylpropionitrile) (AIBN; Sigma-Aldrich, 98%, recrystallized from methanol) was added as an initiator. The mixture was purged with argon for 30 min to remove dissolved oxygen and then heated to 80 °C to initiate polymerization. After 24 h, the reaction was quenched by dilution with 15 mL of Milli-Q water, followed by extractions with hexane twice to remove unreacted monomer and residual DMSO. The pH of the aqueous phase was adjusted to 6–7 using concentrated aqueous HCl. The resulting polymer hydrochloride salt was precipitated by slowly pouring the reaction mixture into 300 mL of acetone. The precipitated solid was collected, redissolved in water, and reprecipitated in acetone for further purification. After allowing the solid to sediment, it was collected, washed with fresh acetone, transferred to a silicone dish, and dried in air at 80 °C for 24 h. A final yield of 3.2 g of purified product was obtained.

The molecular weight and the distribution of the polymer PDMAPMA were estimated through the Gel Permeation Chromatography (GPC) technique, and the results are shown in Figure S1. The sample was analyzed on an Agilent Technologies 1200 series machine using a PSS SPV 5 μm column and PSS SECurity RI detector, calibrated with PEG standards in 0.05M NaNO_3_ eluent. The solid PDMAPMA powder was dissolved in the same eluent to a concentration of 10 mg/mL and subsequently filtered through a 0.45 μm Nylon filter. Ethylene glycol was used as an internal standard for the measurement. The number-average molecular weight of the system (*M*_*n*_) is 7575 Da.

#### Sample Preparation

Aqueous solutions of PDMAPMA at concentrations of 0.1, 0.3, and 1 wt % were prepared by dissolving the polymer in water, followed by sonication in an ultrasound bath. A 1 wt % solution of multiarmed PS–PMAA star-block amphiphilic polyelectrolyte was prepared according to the protocol established by Raffa et al.^36^ Equal aliquots of the star solution and PDMAPMA solutions were combined and vortexed to obtain blends of star polymers with varying linear PDMAPMA content.

#### Shear Rheology

Rheological experiments were conducted using a Discovery Hybrid Rheometer (HR-2) from TA Instruments (United States). All measurements were performed at 20 °C with stainless steel 25 mm diameter parallel plates in a water-saturated atmosphere to minimize water evaporation. Dynamic strain sweeps (DSS) were executed at 100 rad/s to determine the linear and nonlinear viscoelastic regimes. Before testing, the suspensions underwent a rejuvenation and aging protocol to erase mechanical history and attain a steady state condition before any test (see ref 35 for details). The rejuvenation consisted of a dynamic time sweep (DtS) at 1 rad/s and a selected 200% shear strain amplitude (well above the linear viscoelastic regime), typically for 60–100 s, until steady state was reached. The aging was also performed via a DtS conducted at 1 rad/s but at a shear strain amplitude falling in the linear viscoelastic regime for 200 s to build up the internal stress-free structure of the material. Consequently, frequency sweeps were performed over a range of frequencies varying from 100 to 0.01 rad/s.

## Results and Discussion

Figure 1 shows the studied system. We vary the concentration, the charge ratio and the LPE length. The total number of beads and charge neutrality are maintained in all systems. Figure 1a is a simulation snapshot at the end of the production run for an equal mixture of LPEs and counterions at concentration *c* = 0.1 vol %. During the charging process, LPEs form a complex with the SPEs, as shown in Figure 1b. This complex is composed of an SPE (Figure 1c) with negatively charged arms (blue) and a neutral core (red), a positively charged LPE (Figure 1d) and positively charged monovalent counterions (Figure 1e).

**Figure 1:**
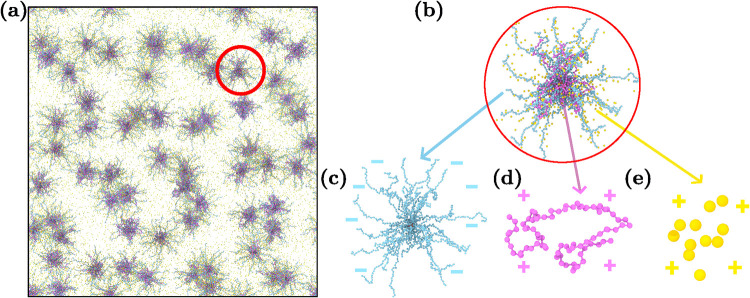
Studied star-linear polyelectrolyte mixture. (a) The simulation box is composed of 64 SPEs with a charge ratio of β = 0.5 at concentration 0.1 vol % in the given snapshot. (b) A spherical complex formed by an SPE, an LPE and counterions. (c) SPE with *f* = 32 arms of length M = 40. The SPE arms (blue) are negatively charged, and the core (red) is neutral. (d) A positively charged LPE of length *L* = 80. (e) Positively charged counterions.

### Single-Molecule Properties

Figure 2a shows the radius of gyration (*R*_g_) of SPEs that is given by

6 $$R_{g}^{2}=\left\langle {\left|{\mathrm{r⃗}}_{i}-{\mathrm{r⃗}}_{\mathrm{COM}}\right|}^{2}\right\rangle$$

where *r⃗*_*i*_ is the position of all beads within a chain and *r⃗*_COM_ is the center of mass position of those beads. Similarly, in Figure 2b, we report the hydrodynamic radius (*R*_h_) as

7 $$\frac{1}{R_{h}}=\left\langle \frac{1}{\left|{\mathrm{r⃗}}_{i}-{\mathrm{r⃗}}_{j}\right|}\right\rangle$$

where *r⃗*_*i*_ and *r⃗*_*j*_ are all possible positions of chain monomers with *i* ≠ *j*.^42^ The brackets denote the ensemble average that runs over all SPEs and time frames in both cases. The size of the SPEs does not change at low *c* upon increased packing. This regime is known as the dilute regime, where SPE sizes are insensitive to changes in the concentration.^26^ The concentration at which SPE size starts shrinking is the overlap concentration (c*).^43^ At concentrations below c*, the SPEs behave independently in solution, and above c*, they begin to interact and compact, marking a transition to more concentrated regimes.^26^ From the theory of star polyelectrolytes in solution, this overlap concentration is expressed as a function of the star’s architectural parameters and is given as

8 $$c^{*}\approx \frac{\mathrm{fM}}{R^{3}}$$

where *f* is the number of arms in the star, *M* is the arm length and *R* is the size of the star at the dilute limit.^26^ Since c* corresponds to the number fraction of SPEs, we convert it to a volume fraction by multiplying it by the volume of a single SPE bead and then express the result as a percentage. If we compute the theoretical *c*_th_^*^ for our systems without LPEs (β = 0), we obtain

9 $$c_{\mathrm{th}}^{*}\approx \frac{32\times 40}{{\left(40.5\right)}^{3}}\times \frac{4\pi }{3}\times 0.5^{3}\approx 1\%$$

**Figure 2:**
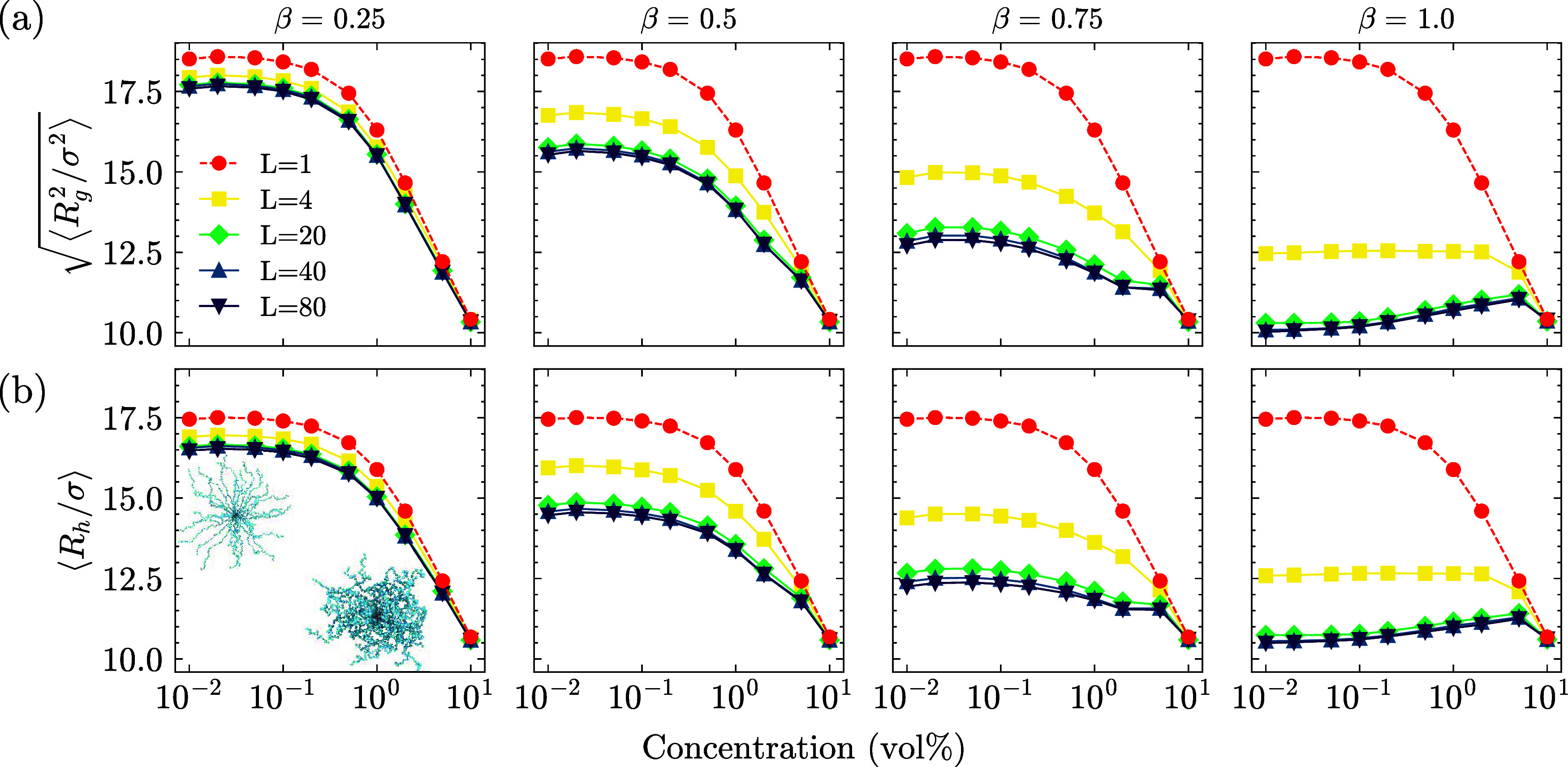
Star polyelectrolyte sizes are measured by two different metrics. (a) The radius of gyration of SPEs is calculated for a range of concentrations at different charge ratios (β) with different LPE lengths. Red data points with dashed lines indicate the reference case where the charge ratio is 0, and the system is entirely composed of counterions. Increasing packing shrinks the SPEs, with the shrinkage being more prominent with increased amount and length of LPEs. At high charge ratios (β = 1.0), the SPEs collapse at low concentrations and start expanding in the presence of long LPEs (*L* > 4) up to concentration 5%. However, the further increase in the concentration makes them shrink and become comparable to the reference case. This suggests a competing effect between high packing and electrostatic forces, which we will investigate further. (b) The hydrodynamic radius of SPEs shows a similar trend as the *R*_g_. The inset snapshots show single SPE architectures at *c* = 10^–2^% (left snapshot) and at *c* = 10% (right snapshot) for β = 0.0.

Since the theory assumes that the stars are fully stretched at low concentrations neglecting thermal fluctuations, we use *R* = 40.5 in the above equation. This prediction is slightly off compared to our data, which shows that SPEs start shrinking at c* = 0.5% (Figure 2, red circles), although the significant change is at around *c* ≈ 1% for a pure SPE solution. A more detailed characterization of the structure and rheological properties of pure SPEs has been performed by us in a recent work.^35^ While c* is formally affected by the introduction of LPEs, we show in the following that all c* values remain within the same order of magnitude for all our systems, as long as the fraction of LPEs is low enough to not trigger new collective behavior.

Using linear polyelectrolytes as additives significantly affects the star size. Adding more LPEs shrinks the SPEs in the system at a given concentration for all LPE lengths, and the degree of shrinkage becomes more pronounced as the LPE length increases. Longer linear chains experience a smaller entropic penalty upon condensation, making them more likely to localize within SPEs. The ratio of the hydrodynamic radius to the gyration radius (*R*_h_/*R*_g_) is reported in Figure 3, a quantity used to capture the changes in the shape of the polyelectrolytes. The theoretical value of this quantity at the dilute limit for SPEs is ∼0.93,^44^ which we recover with simulations.

**Figure 3:**
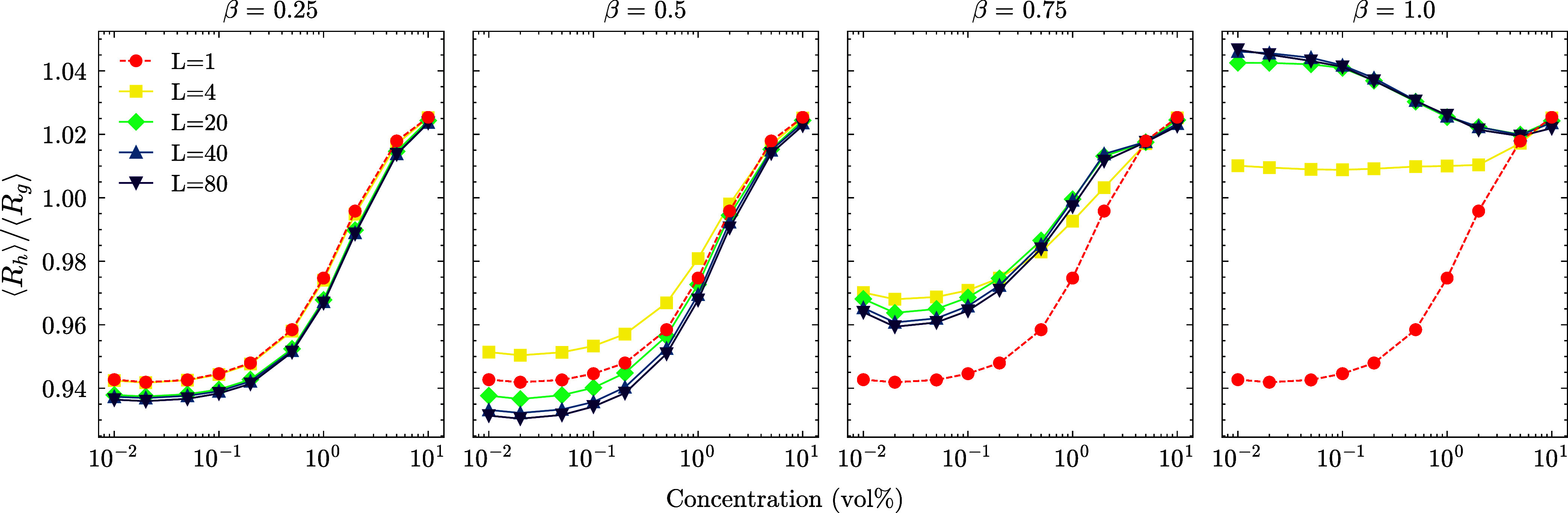
Ratio of the average hydrodynamic radius to the radius of gyration for star polyelectrolytes. This quantity is used to differentiate between various polymer conformations: ∼0.79 for an ideal Gaussian coil and ∼1.29 for a uniform sphere.^45,46^ It is independent of the concentration at the dilute limit. Increasing β increases the ratio, and for the intermediate values of β, the ratio decreases with increasing LPE length.

SPEs become smaller and converge to a uniform sphere limit with increasing concentration for charge ratios β < 1.0 in agreement with a previous study for stars with a moderate number of arms (β = 0.0).^30^ In the case of an equal SPE-LPE mixture (β = 1.0), this trend is reversed for long LPEs *L* > 4. They are closer to the uniform sphere limit at low concentrations and become more aspherical with increasing concentration. In all cases, the ratio converges to ∼1.025 at the highest concentration. Furthermore, both the *R*_g_ and *R*_h_ converge to the same respective values at the extreme packing. This reflects the competition between the high packing density and electrostatic interactions, with the extreme packing ultimately dominating, compressing the SPEs as if no LPEs were present. The most striking observation is the unexpected increase in SPE size after reaching the overlap concentration at the same high β and long *L* limit, already starting at *c* ∼ 0.1% (below the overlap concentration) and peaking after reaching the pure SPE overlap concentration. This counterintuitive effect and the crossover point in the *R*_h_/*R*_g_ ratio are explained by variation in the net charge within the star’s imaginary bounding sphere, as shown in Figure 4, which is also related to a phase change of the system, which we discuss in the following section. At fixed SPE concentration, increasing β always leads to a decrease of the SPEs’ *R*_g_ values. In Figure 4, we find the sphere with the smallest radius that encapsulates each SPE, then count all charge-bearing particles within this sphere, including the negative contribution from the stars’ arms. The resulting number is the net charge of the sphere, and we normalize this value by the total number of charges that an SPE carries, i.e., *fM*. A negative value indicates that the effective spheres carry a net charge, and 0 indicates a fully neutralized sphere. In the case of overlapping spheres, we attribute the charges to a sphere with the closest star core.

**Figure 4:**
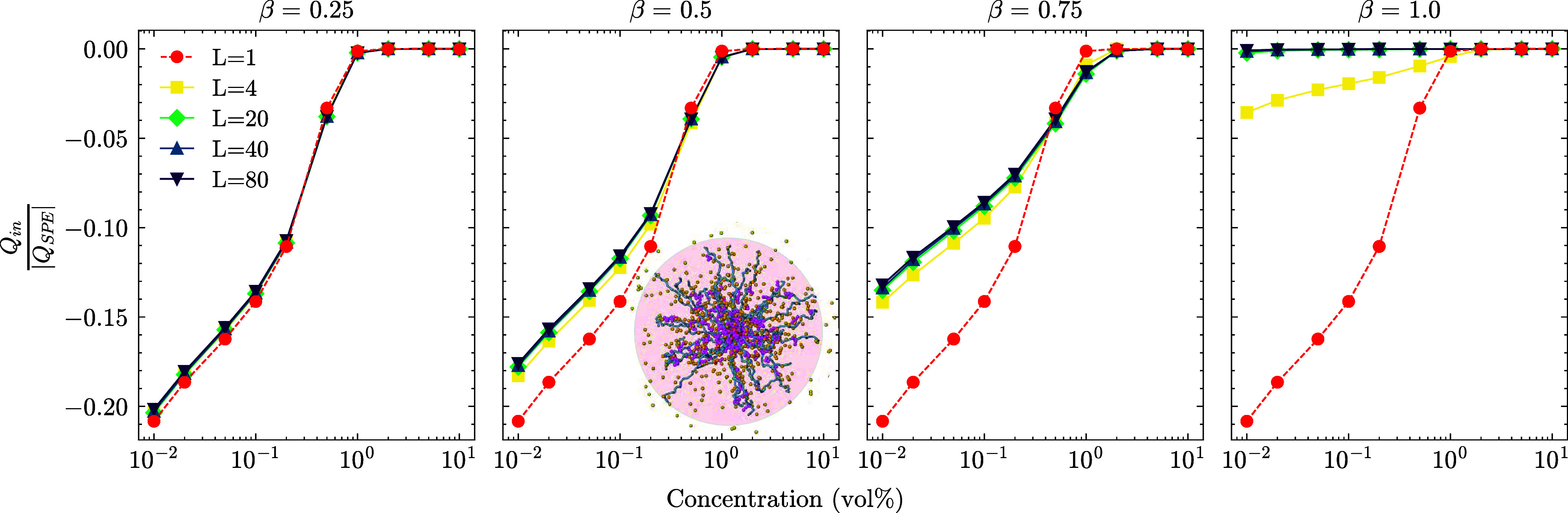
Net charge within the bounding sphere of SPEs normalized by the absolute SPE charge. The inset snapshot illustrates the definition of a bounding sphere for SPEs. The particle sizes are adjusted for visual purposes and not to scale. A negative value indicates that the bounding sphere of SPEs carries an effective negative charge. As this value converges to zero, this sphere carries no effective charge and is neutralized. In the case of overlapping spheres, the charge is assigned to the sphere with the closest SPE core to avoid double counting and maintain charge neutrality in the system. The effective charges in the case of long LPEs at low β contribute to the effect of increased LPE size. The spheres that carry an effective charge attract the LPEs and make them expand. These LPEs form a bridge between two SPE cores, as shown in Figure 7.

Added LPEs increase the number of charges trapped in the SPE bounding sphere. All our systems are fully neutralized around the theoretical overlap concentration (*c* ≈ 1%) of the pure SPE solution. With an increasing charge ratio, neutralization occurs even at lower concentrations for long chains (*L* > 4). Despite having the same total number of positive ions at a given β, the neutralization effect is profoundly different based on the length of these species. The significance comes from the entropy of the positively charged and connected species. The collective motion of positive ions in a longer chain neutralizes the SPE bounding spheres even at the lowest concentration. In contrast, the contribution of counterions is more sensitive to the concentration (see Figures S2–S3 for the contribution of counterions and LPEs to the net charge, respectively). On average, some SPE spheres are neutral, and some carry an effective negative charge. The size of the LPEs is also affected by LPE length and concentration, as well as SPE concentration. We measure the *R*_g_ of LPEs in Figure 5.

**Figure 5:**
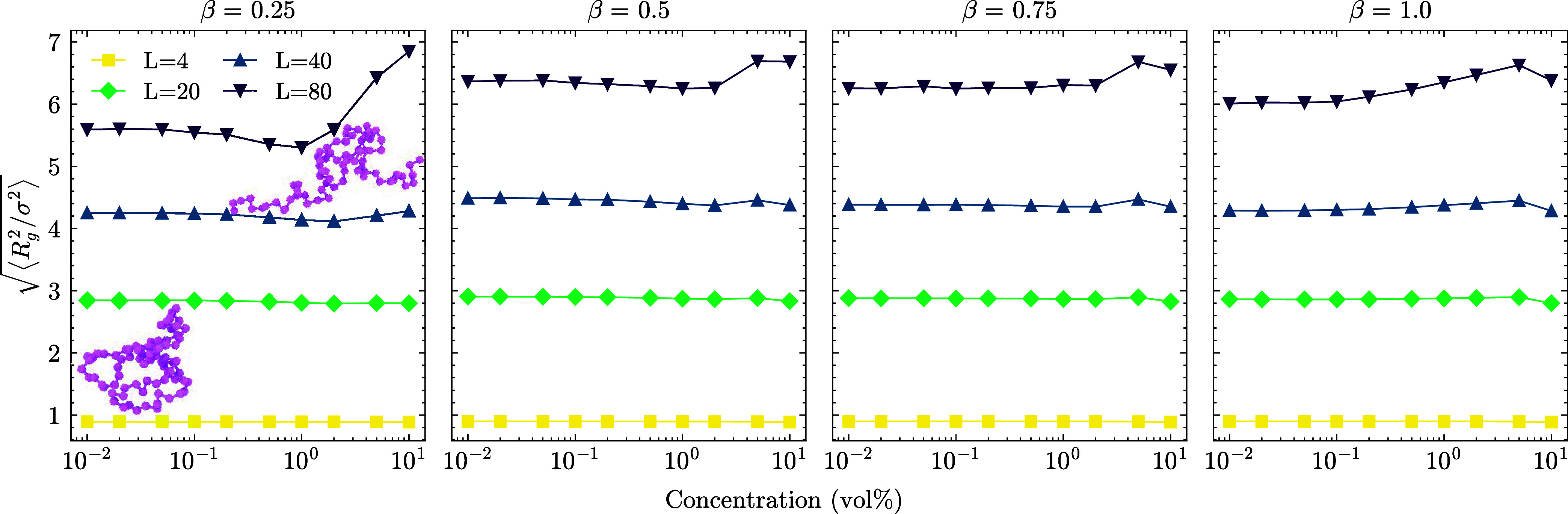
Radius of gyration of linear polyelectrolytes calculated for various concentrations at different charge ratios. Short chains (*L* < 40) are not affected by the charge fraction; only *L* = 20 at high β shrinks with the effect of extreme packing. There is a transition point at *L* = 40, which is the arm length of SPEs, where LPEs start expanding at low β. We attribute this behavior to the effective charge of SPE arm beads that do not have bound counterions (see Figure 6) and can attract and stretch long chains between two micelles at high SPE concentration. The inset shows two snapshots of an LPE of length *L* = 80 around an SPE core at *c* = 0.01% and *c* = 10%, respectively.

For fixed LPE length (*L* = 80), the size is most affected at low β due to global and local fluctuations of charges. At the lowest charge ratio (β = 0.25), the LPEs shrink upon packing up to the overlap concentration, after which they expand. There exist few LPEs compared to higher charge ratios which is insufficient to neutralize all SPEs; therefore, some SPE spheres carry an effective charge while others, on average, are neutral. Even at high concentrations when the bounding sphere of the SPE is on average neutral, monomeric counterions are entropically less likely to be bound to the SPE arms, leaving local charges that can attract long LPEs. We can quantify the ratio of SPE charges screened by counting the positively charged beads condensed along the SPE arms as reported in Figure 6.

**Figure 6:**
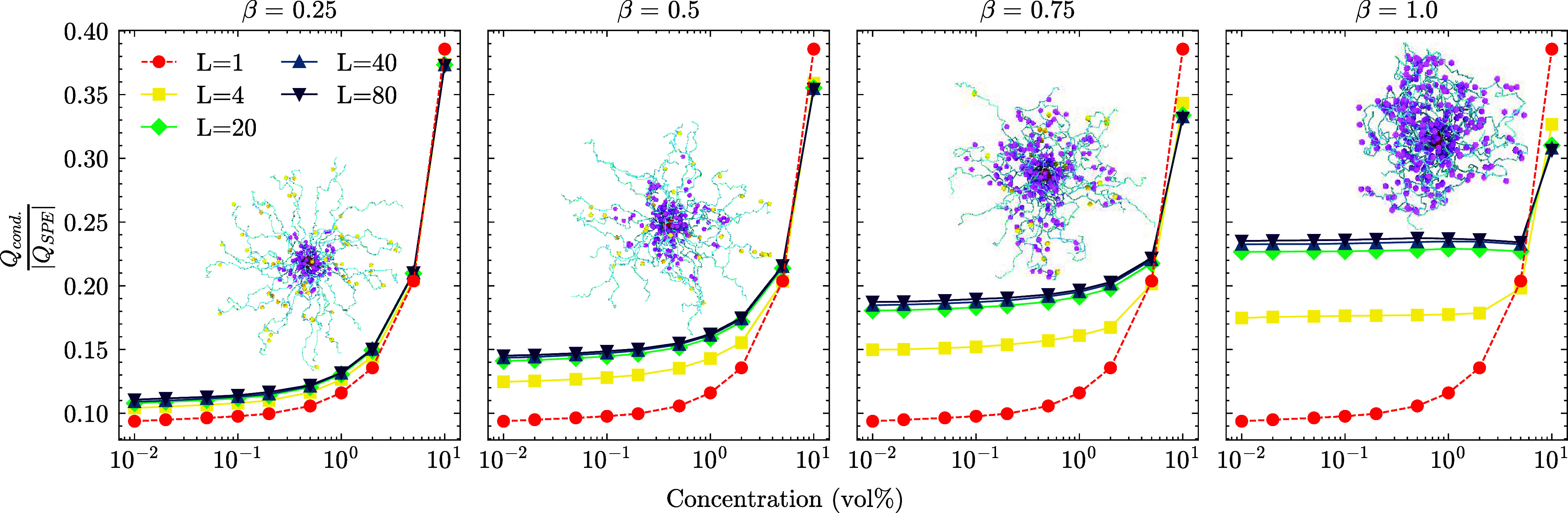
Ion condensation along the star arms normalized by the absolute star charge. Inset snapshots show a single star and positively charged ions that are condensed on its arms for *L* = 80 system at each β. The particle sizes are adjusted for visual purposes and not to scale. The number indicates the ratio of star beads that are electrostatically screened by the oppositely charged species. The screening effect increases with the increasing LPE length for a given charge ratio and with increasing charge ratio for a given LPE length. We measure the condensation by a similar contact analysis for the trapped ions with a cutoff distance of $2^{1/6}\sigma$ , corresponding to the distance the LJ potential attains its minimum.

Where *Q*_cond._ is the number of positively charged species condensed on the SPE arms. We measure this quantity by a similar contact analysis to the one in Figure 4. In this case, we consider an ion condensed if it is in contact with an SPE bead within a cutoff radius of $2^{1/6}\sigma$ , the distance where LJ potential attains its minimum. Within this cutoff, positively charged ions are attached to the SPE arms, and their interactions with other charged species are screened. As a result, positively charged LPEs are attracted to a charge-carrying SPE sphere from a neutral one. This attraction influences not only individual LPE conformations but also gives rise to distinct collective behaviors in the system.

### Collective Properties

One notable outcome is the formation of bridge-like structures, where the LPEs stretch and span between two SPE cores, increasing their size. We refer to such LPEs as bridge-forming chains. These chains can be quantified through a straightforward contact analysis, as shown in Figure 7.

**Figure 7:**
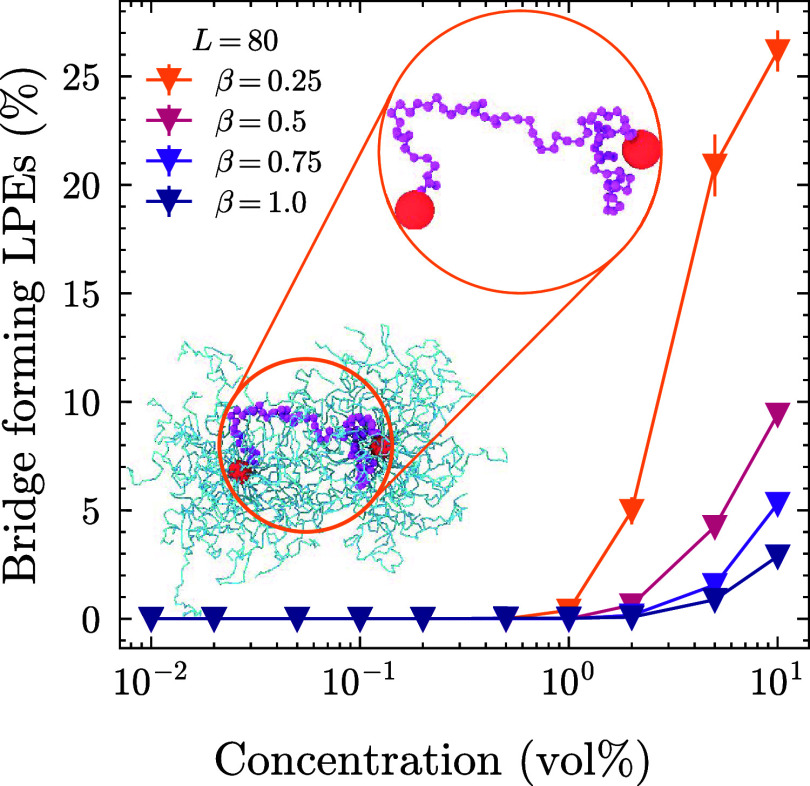
The effect of charge ratio to the percentage of bridge forming LPEs at a given LPE length. When an LPE extends from one SPE core to another, we classify it as a bridge chain. The bridge formation is observed for the longest LPE (*L* = 80). The effect is more pronounced in the case of low charge ratios. This is caused by the lower condensation of positive charges along the SPE arms at low β, see Figure 6. See the SI for the effect of shorter LPEs *L* < 80. The snapshot shows a bridge chain between two SPE cores at β = 0.25. A zoomed-in version without the SPE arms is also given. Particle sizes are adjusted for visual purposes and are not proportional to actual sizes.

When two beads belonging to an LPE come into contact with at least two SPE cores simultaneously, we consider it a bridge-forming chain. The contact cutoff in this analysis is set to 3σ the additive distance of the LJ cutoff 2.5σ and the LPE bead radius 0.5σ. We show the bridge formation for *L* = 80 system at different β (see the remaining systems in Figure S4). As we speculated previously, when the number of LPEs is insufficient for the full neutralization of the system and condensation along the SPE arms is less pronounced (β = 0.25), the bridge formation is at its maximum, whereas for increasing β, bridge formation is suppressed as more SPEs are effectively neutralized and their charges are further screened. This prevents one SPE sphere from attracting an LPE. The bridging also influences the macroscopic properties of the solution by causing a liquid–liquid phase separation, meaning that the homogeneous SPE-LPE solution is divided into two phases with different compositions. For polyelectrolytes, this phenomenon is known as complex coacervation, where one region in the solution is rich in oppositely charged polymer complexes, called a coacervate phase, and another region is poor in polyelectrolytes and contains primarily the solvent, called the supernatant phase.^47–49^ We show the phase separation in Figure 8 by measuring the spatial distribution of the SPE cores, the radial distribution function *g*_core‑core_(*r*), for *L* = 80 with their corresponding snapshots at the end of the equilibration step.

**Figure 8:**
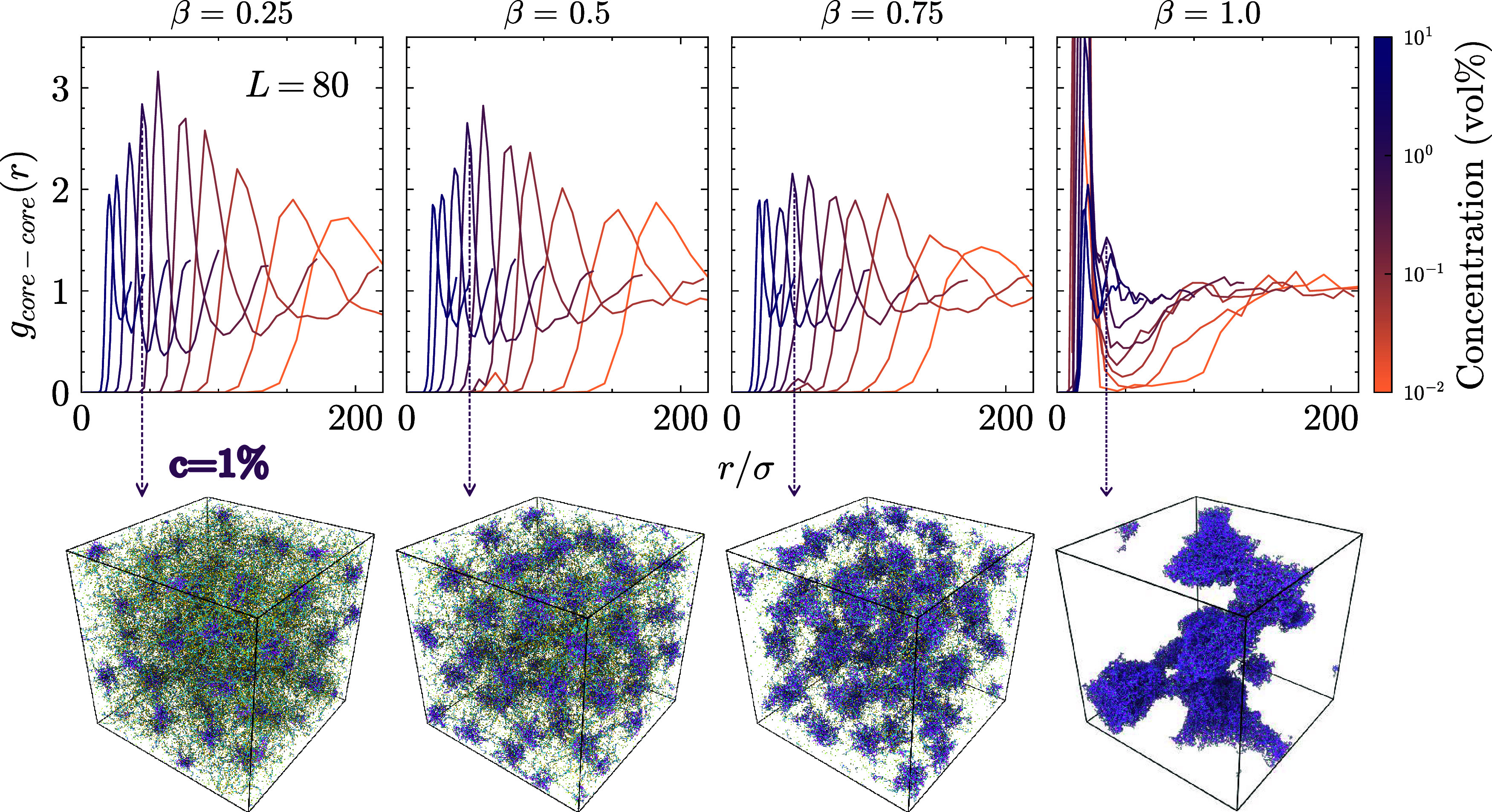
The radial distribution function of the star cores for the system with *L* = 80 LPEs. The strong signal at β = 1.0 indicates high localization of SPEs in space. The corresponding snapshot visualizes this localization and shows the complex coacervation. For β < 1.0, there are no strong *g*(*r*) peaks, suggesting a liquid-like behavior and the corresponding snapshots show the liquid structure. An interesting observation is the structural reentrance that these systems undergo. The snapshots are taken at the final simulation frame at concentration *c* = 1%.

We observe the phase separation at high charge ratios and for long chains in the solution. SPE cores are highly localized in space at β = 1.0, characterized by the strong first peak of the *g*(*r*), and the corresponding snapshot shows a fully phase-separated system. Although there is no strong signal in the *g*(*r*), we can visually detect the formation of small droplets for β = 0.75. Further studies focusing on the phase separation of these mixtures could verify the value of critical β and concentration where the transition takes place. A preliminary phase diagram based on the parameters currently investigated is reported in our SI, see Figure S7. The solution remains liquid-like for all other β values. The phase behavior of the solution also depends on the length of the LPEs. In Figure 9 we show the *g*(*r*) of the SPE cores for the shortest LPE length *L* = 4.

**Figure 9:**
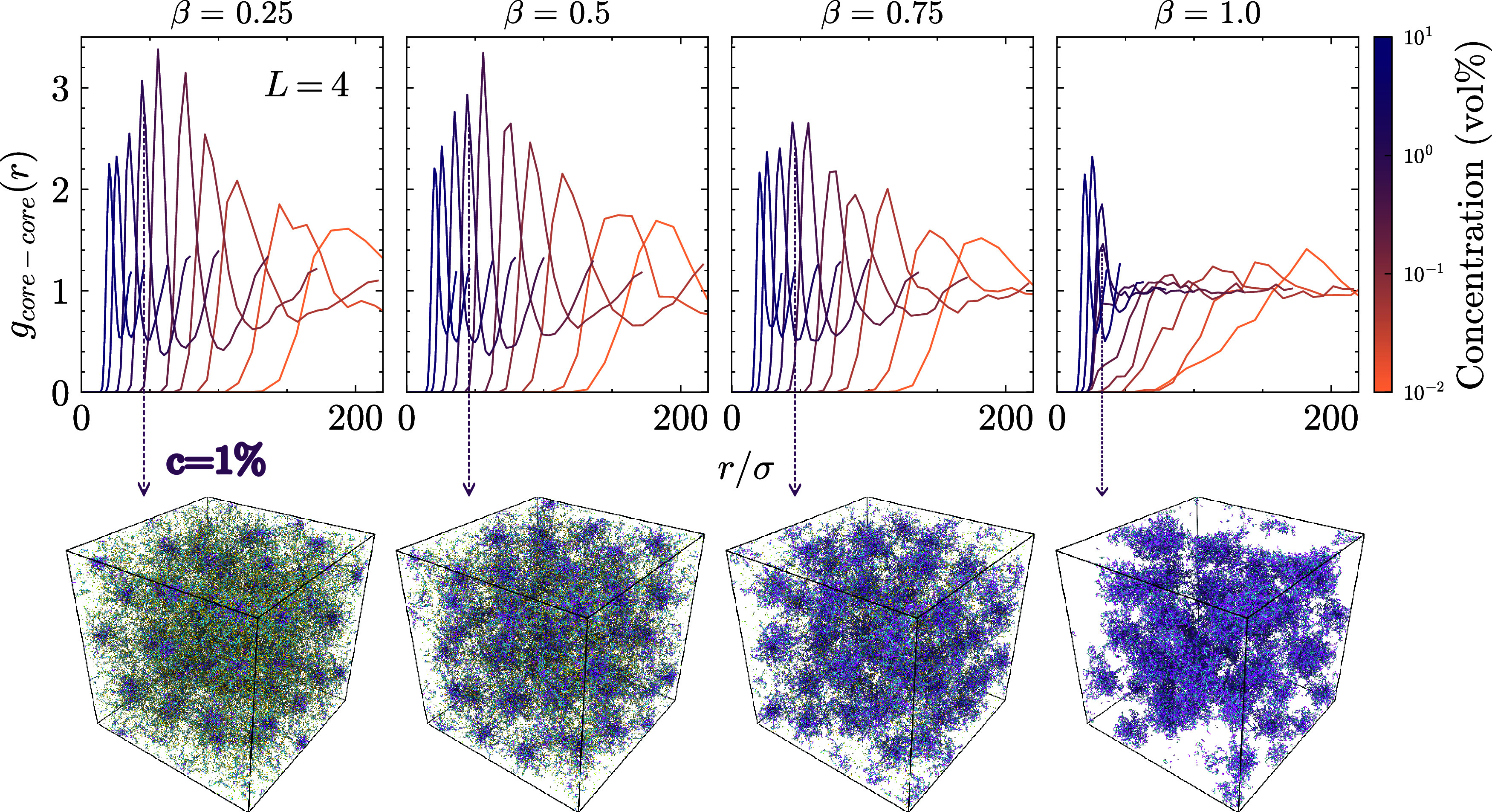
The radial distribution function of the star cores for the system with *L* = 4 LPEs. The snapshots show the homogeneity in the system with increasing β at concentration *c* = 1%.

At low β, the solution remains liquid-like and at β = 1.0 we start to observe the droplet formation without a full phase separation. The phase separation only occurs for long chains with a high charge ratio, and the remaining systems have liquid-like behavior. See the phase separation for *L* = 20, 40 in Figures S5–S6.

An important characteristic of these systems is their ability to exhibit structural reentrance without any corresponding dynamical phase reentrance (See Figure S12 for the MSD of the star cores). All homogeneous systems shown in Figures 8–9 demonstrate this structural reentrance, which is identified by analyzing the first peak of the *g*(*r*). The peak shifts to the right as concentration increases, reflecting greater core–core distances. At low concentrations, the correlation strength at a given distance is weak, as indicated by the low height of the *g*(*r*) peak. The peak positions and their values are given in Figures S8–S11. The correlation strength grows with increasing concentration, reaching a maximum at the overlap concentration c* = 0.5%. Beyond this point, as concentration continues to increase, spatial correlations weaken, and the peak height of *g*(*r*) diminishes, returning to values similar to those observed at low concentrations. This return to the initial state defines the structural reentrance, which has also been observed in other charged systems such as charged microgels.^50^ Note that in the regime where liquid–liquid phase separation is observed, the time scales needed to reach an equilibrated structure go beyond our simulation time scales, so that the phase-separated structures shown in Figure 8 might still evolve toward equilibrium. While this remains an interesting point of study for future works, here we simply claim that the right composition of the stars shifts from a collective glassy behavior (due to effective repulsion) to a collective phase separation (due to effective attraction), as we can also qualitatively validate experimentally.

### Experimental Validation of Phase Separation

Finally, we demonstrate how this microscopical change influences macroscopic solution properties by presenting experimental data of qualitatively similar systems (see also our previous work for additional details ref 35). In Figure 10, we report the linear viscoelastic spectra of SPE solutions containing additive LPEs, along with their complex viscosity. Our findings indicate that a small fraction of LPEs reduces the viscosity of the system, compatibly with the SPEs size reduction observed computationally. Further increasing the concentration of LPEs (represented by β in simulations) ultimately drives the system to phase separate at sufficiently high LPE concentrations, with visible liquid–liquid phase separation (inset pictures in Figure 10b) and a significant increase in viscosity of the coacervate (measured after removing the supernatant phase). Thanks to our previous findings,^35^ these rheological observations allow us to infer variations in the structure of the SPEs in line with the simulated results.

**Figure 10:**
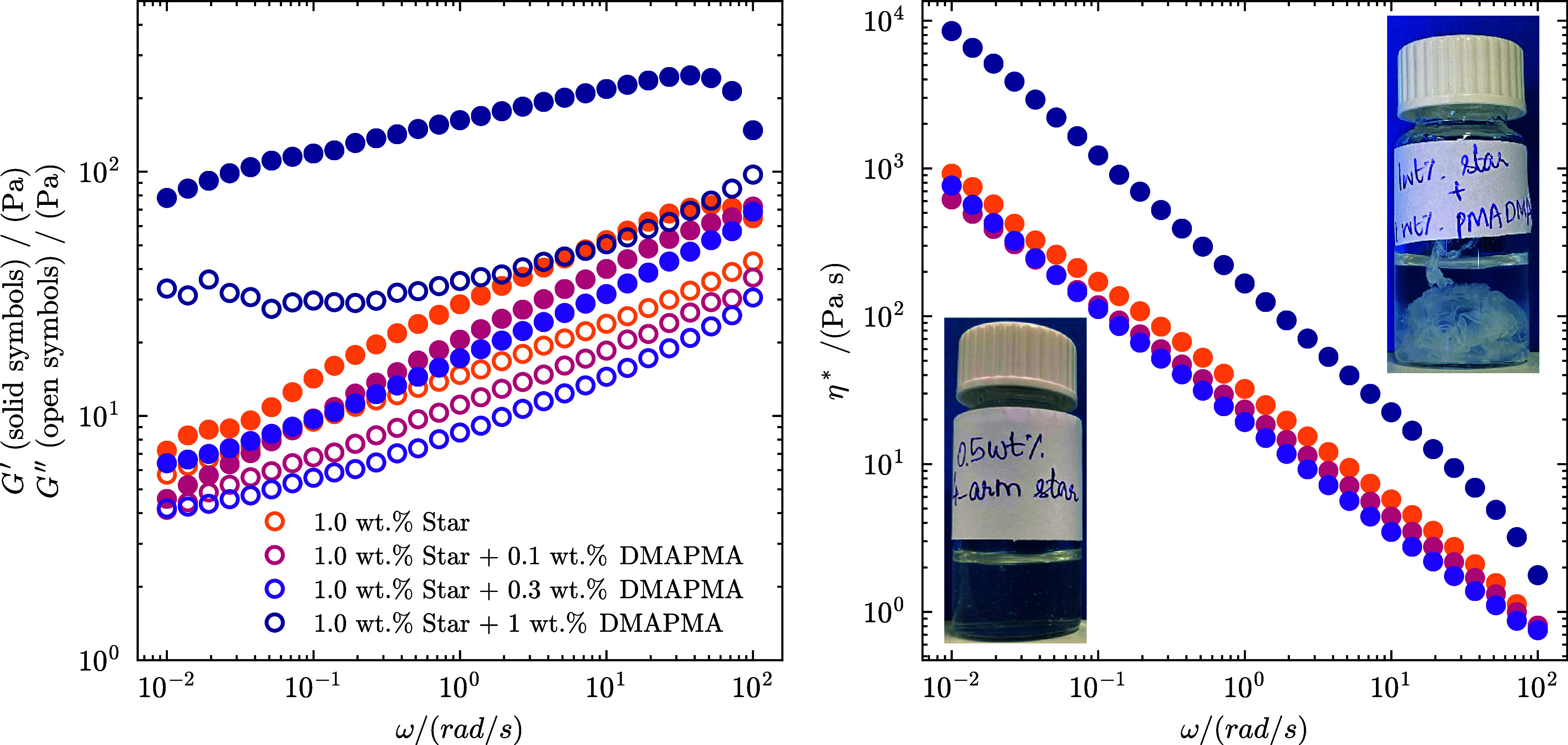
Linear viscoelastic spectra expressed in terms of (a) storage modulus *G*′ (solid symbols) and loss modulus (open symbols), (b) complex viscosity η*, as a function of oscillation frequency ω for multiarmed PS–PMAA star polyelectrolyte solutions and its mixture with positively charged *N*-[3-(Dimethylamino)­propyl]­methacrylamide (DMAPMA) across varying concentrations. The insets in (b) depict the homogeneous PS–PMAA star polyelectrolyte solution without DMAPMA (lower snapshot) and the phase-separated system upon the addition of 1 wt % of DMAPMA (upper snapshot).

We remark that scaling down the MD model in this work to allow the study of a large parameter range means that, compared to our previous work, the MD and experimental systems are not mapped 1:1. The experimental micelles have longer arms (∼367 monomers per arm) with lower grafting density and longer linear chains (∼345 monomers per chain). Therefore, we do not expect to use the experimental data to pinpoint the critical conditions for phase separation quantitatively, but rather to demonstrate that the change in viscosity and visual observations reflect the observed behavior of the model systems, in which higher LPE concentration first shrinks the SPEs and ultimately leads to liquid–liquid phase separation.

## Conclusions

We have investigated mixtures of highly charged star polyelectrolytes (as model micelles) with positively charged linear polyelectrolytes across varying concentrations, charge ratios, and chain lengths. Our simulations combined with experimental observations on similar block copolymer micelles with a highly charged corona reveal that electrostatic interactions, packing effects, and chain connectivity dictate SPE structural properties and the overall phase behavior and viscosity of the system.

At dilute concentrations, the SPEs remain fully stretched until the overlap concentration is reached. Beyond this point, enhanced intermolecular interactions lead to the shrinking of SPEs. Adding LPEs further modulates this behavior: even relatively small amounts of LPEs can induce partial or complete neutralization of the effective star spheres, generating local charge imbalances. These imbalances lead to bridging chains, in which a single LPE extends between two SPE cores, thereby altering both the SPE and LPE conformations. In particular, longer LPEs have a greater tendency to form bridges between stars, which intensifies local clustering.

The system exhibits phase separation at higher charge ratios and with sufficiently long LPEs, forming a polyelectrolyte-rich phase alongside a dilute supernatant phase. Shorter LPEs, on the contrary, predominantly yield homogeneous mixtures with less pronounced bridging. In addition, we observe a structural reentrance phenomenon in which the core–core correlations of the SPEs initially strengthen with increasing concentration and then weaken after surpassing the overlap concentration. Experimental rheological data confirm that raising the LPE content eventually drives the mixture to phase separation, aligning closely with the simulation results.

Overall, these findings highlight how concentration, chain length, and charge ratio collectively dictate the structural and phase behavior of SPE–LPE mixtures. The insights gained here guide the tailoring of polyelectrolyte blends in applications that require controlled complexation or responsiveness, such as drug delivery and the design of advanced functional materials.

Building on these findings, several directions remain for further investigation. First, a more systematic study of liquid–liquid phase separation in these mixtures is needed, focusing on the critical concentration and charge ratio thresholds for coacervation to elucidate the underlying equilibrium thermodynamics. Second, the dynamics of pure SPE solutions and SPE–LPE mixtures remain insufficiently characterized, partly due to the extended equilibration times required for such simulations. Third, there is scope for investigating the impact of counterion size and valency on the conformation and phase behavior of SPE solutions, particularly in settings where higher-valence ions could strongly modify electrostatic screening. Finally, systematically examining weakly charged SPEs would offer valuable insight into how partial ionization modifies the conformation, solution phase, and rheological properties. Together, these directions will deepen our understanding of star polyelectrolyte mixtures and guide the design of advanced soft materials.

## Supplementary Material



## Notes

Average molecular weight of PDMAPMA, contribution of positively charged counterions/LPEs to the net SPE bounding sphere charge, percentage of bridge-forming linear polyelectrolytes for all systems, evaluation of the bridge formation with different cutoff radii, radial distribution function of the star cores for all systems, phase diagram of the studied systems, dependence of the position and magnitude of the first peak in the radial distribution function on concentration for all systems, mean squared displacement of SPE cores (PDF)

## Notes

**Code Availability** In an effort to promote Open Science practices, the LAMMPS code used to perform our simulations is available on the GitHub page of our group https://github.com/giuntoli-group/star-polyelectrolyte-solutions
