# Supplementary material for: Characterizing the Structural Conformation of Highly Charged Star-Linear Polyelectrolyte Mixtures in Solution
Source: Macromolecules. 2025 Sep 16;58(19):10464–74. doi: 10.1021/acs.macromol.5c01180 (PMC12530052; doi:10.1021/acs.macromol.5c01180)
Supplement: Supplementary file 1 [file ma5c01180_si_001.pdf]

Supplementary Information: Characterising the structural conformation of highly charged star-linear polyelectrolyte mixtures in solution

Utku Gürel,† Ilija A. Gjerapić,† Wouter J. H. Arends,† Roshan Akdar Mohamed Yunus,‡ Aleksander Guzik,‡ Patrizio Raffa,‡ Daniele Parisi,‡ and Andrea Giuntoli*,†

†*Zernike Institute for Advanced Materials, University of Groningen, Nijenborgh 3, 9747AG Groningen, The Netherlands*

‡*Department of Chemical Engineering, Engineering and Technology Institute Groningen, University of Groningen, Nijenborgh 3, 9747AG, The Netherlands*

E-mail: a.giuntoli@rug.nl

The molecular weight and the distribution of PDMAPMA

Fig. S1 shows the molecular weight (M_w) distribution of the polymer PDMAPMA, which was estimated through the Gel Permeation Chromatography (GPC) technique.

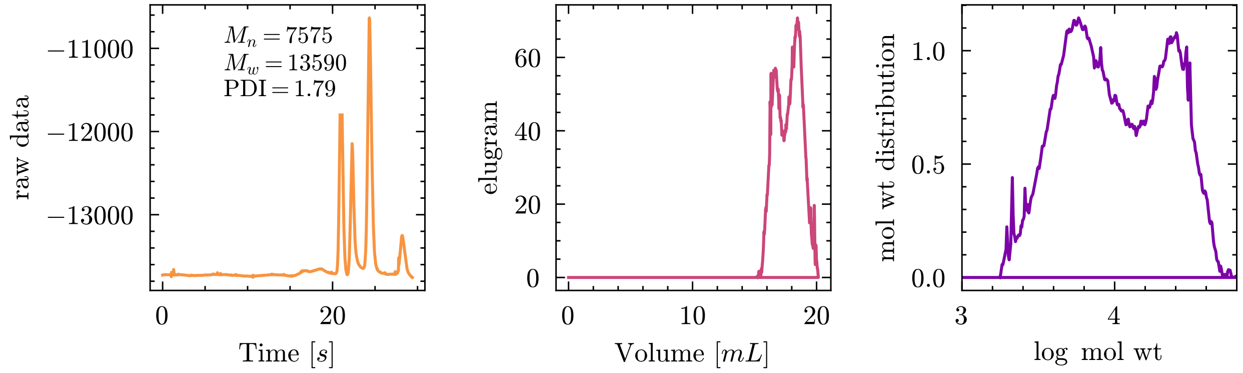

Figure S1: Gel Permeation Chromatography (GPC) chromatograms in determining the average molecular weight of the polymer PDMAPMA.

Contribution of the positively charged species to the neutralisation

Fig. S2 shows the total positive charges contributed by counter-ions within the bounding sphere of an SPE, normalised by the total SPE charge. As the case $\beta = 1$ represents a pure SPE-LPE mixture, no counter-ion contribution is reported.

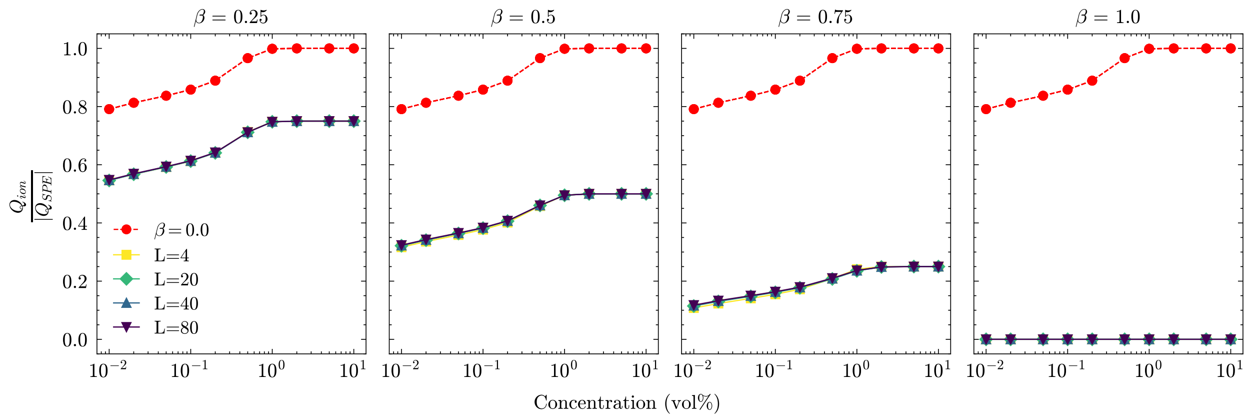

Figure S2: Contribution of positively charged counter ions to the net SPE bounding sphere charge.

Similarly, Fig. S3 shows the total positive charges contributed by LPEs within the SPE

bounding sphere, again normalised by the total SPE charge. At $\beta = 0$, the value is zero, as no LPEs are present in the system.

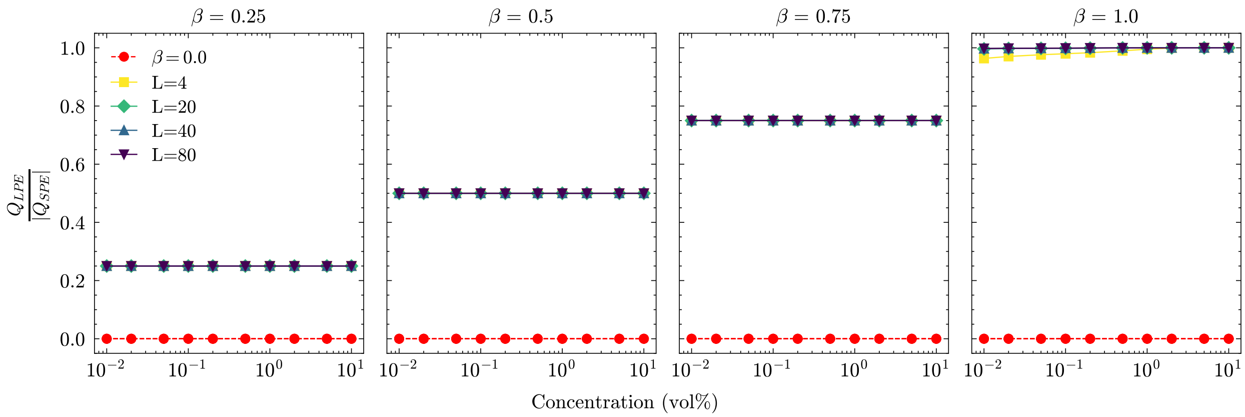

Figure S3: Contribution of positively charged linear polyelectrolytes to the net SPE bounding sphere charge.

Percentage of bridge-forming chains in each system

We report the percentage of bridge-forming LPEs across all investigated systems in Fig. S4. The bridge formation is most pronounced for longer LPEs, as highlighted in the main text. We additionally present the corresponding quantity at an increased cut-off distance $r_c = 5\sigma$ and observe similar trends.

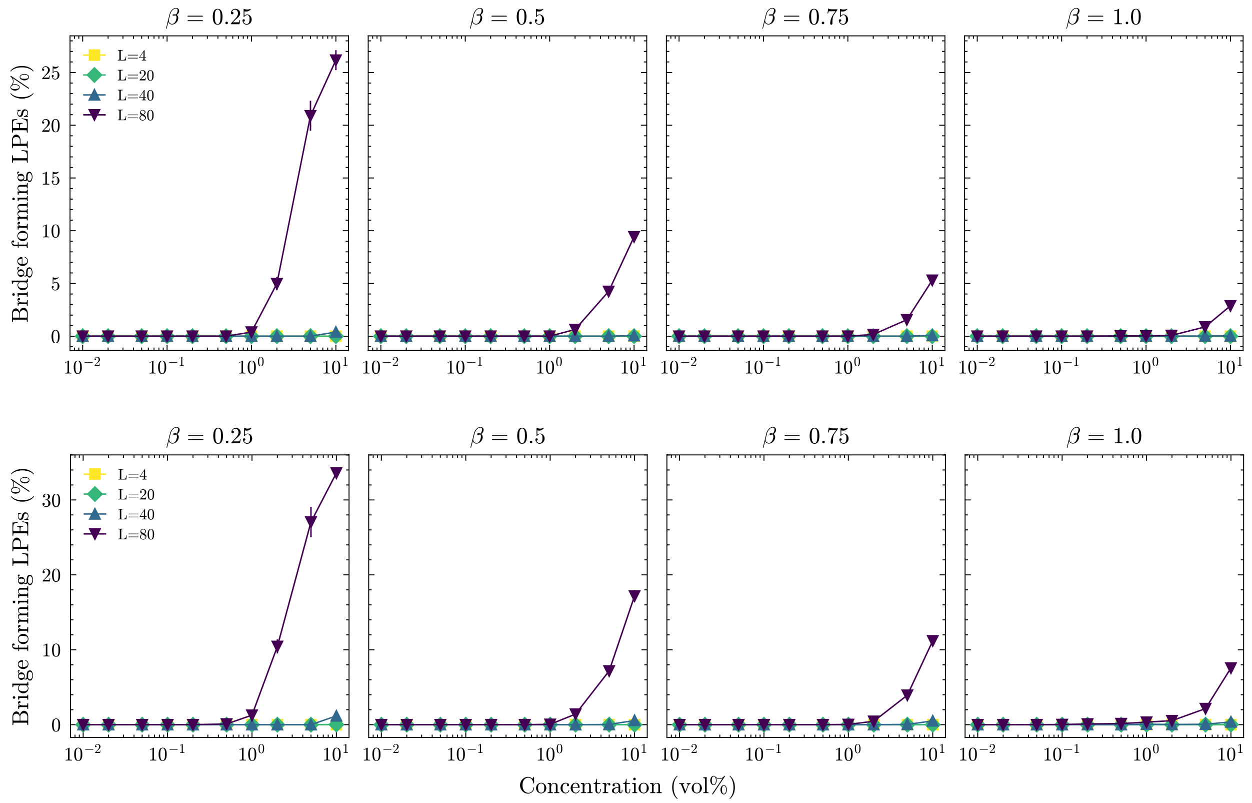

Figure S4: **Percentage of bridge-forming linear polyelectrolytes**. The top row shows the values for all systems examined. The bottom row shows the corresponding values obtained using an increased cutoff radius $r_c = 5\sigma$

Phase separation for long chains

Phase separation is induced by the presence of oppositely charged long linear chains. While we have focused on the longest LPEs in the main text, here we demonstrate that LPEs with intermediate lengths (20 and 40) also drive phase separation. The corresponding radial distribution functions and representative simulation snapshots are provided in Figs. S5-S6.

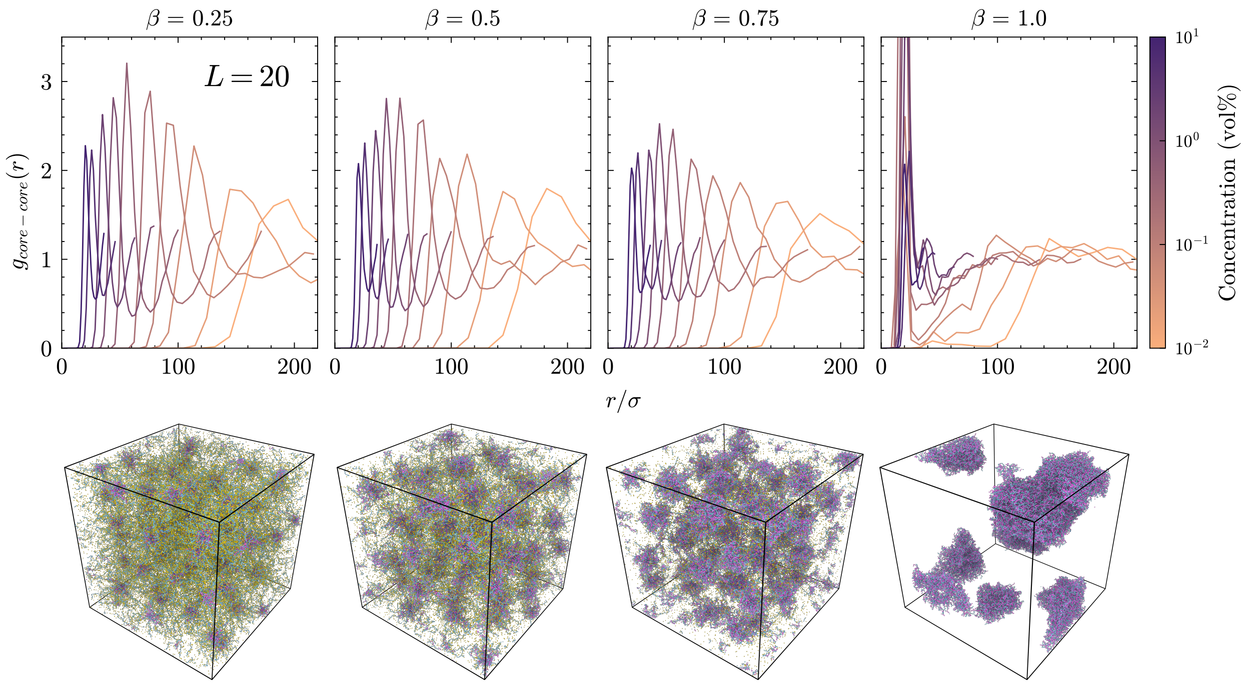

Figure S5: **The radial distribution function of the star cores for the system with $L = 20$ LPEs.** The snapshots show the homogeneity in the system with increasing β at concentration $c = 1\%$.

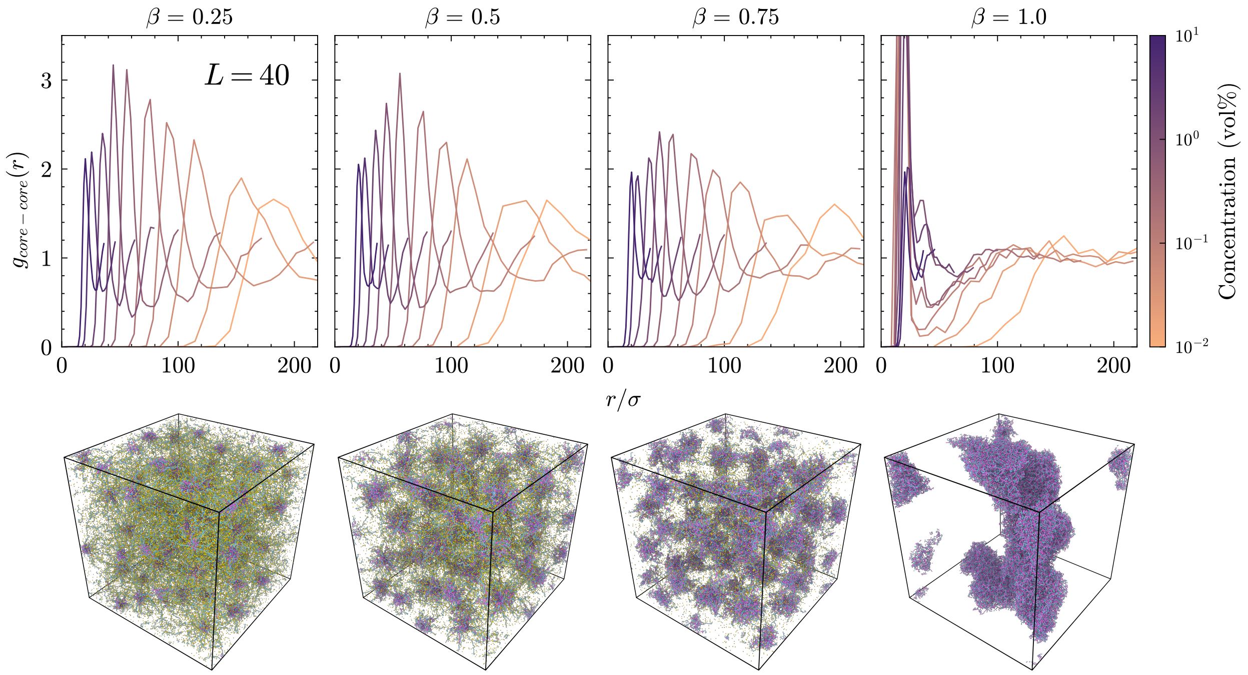

Figure S6: **The radial distribution function of the star cores for the system with $L = 40$ LPEs.** The snapshots show the homogeneity in the system with increasing β at concentration $c = 1\%$.

We show the individual phases of our systems in a 2D phase diagram in Fig. S7.

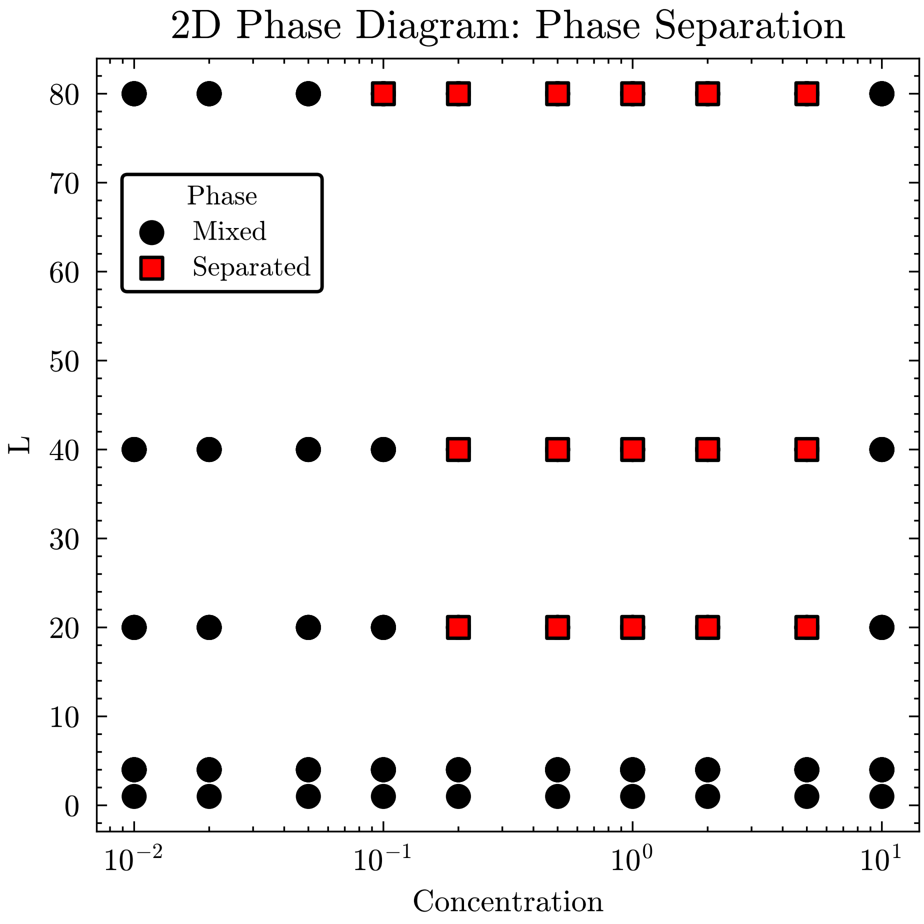

Figure S7: **Phase diagram of the studied systems at $\beta = 1.0$.** Black points show the mixed, and red points show the phase-separated systems in our parameter space. All systems with $\beta < 1.0$ do not show phase separation.

Concentration-dependent structural reentrance

We observe a structural reentrance in these systems as characterised by the $g(r)$ peaks as mentioned in the main text. Here we show these peaks explicitly with their corresponding peak positions in Figs. S8-S11.

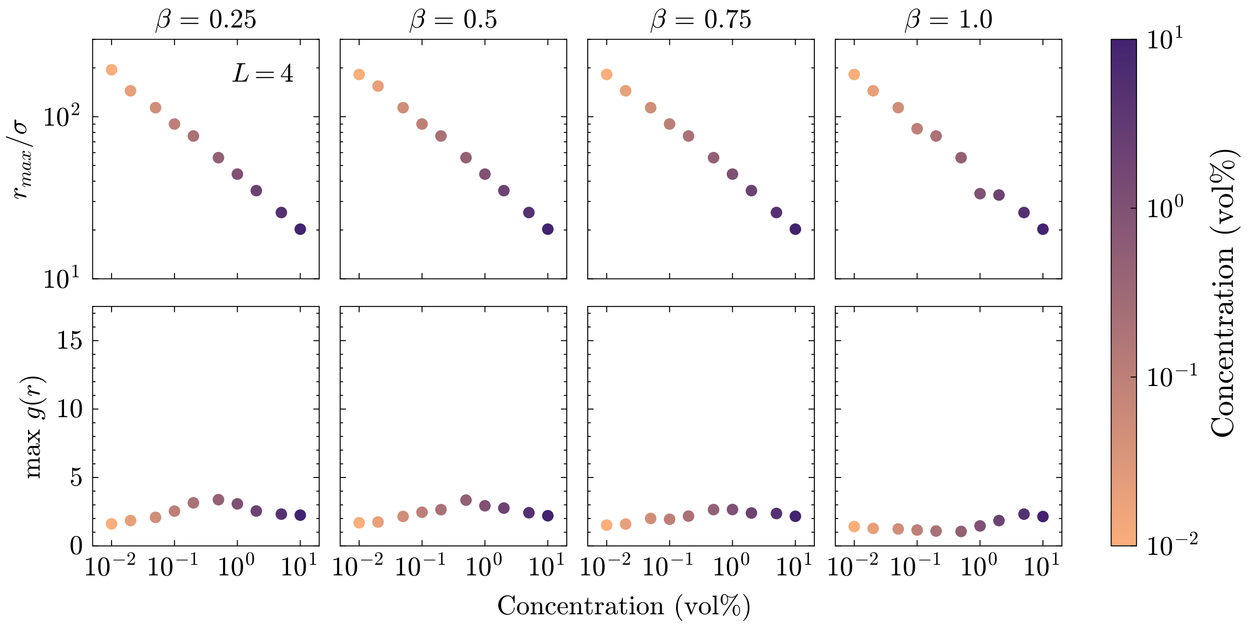

Figure S8: **Dependence of the position and magnitude of the first peak in the radial distribution function $g(r)$ on concentration for various values of β at $L = 4$.** The top row shows the location of the maximum in $g(r)$ as a function of concentration. The bottom row shows the corresponding maximum value of $g(r)$. Colours indicate concentration on a logarithmic scale.

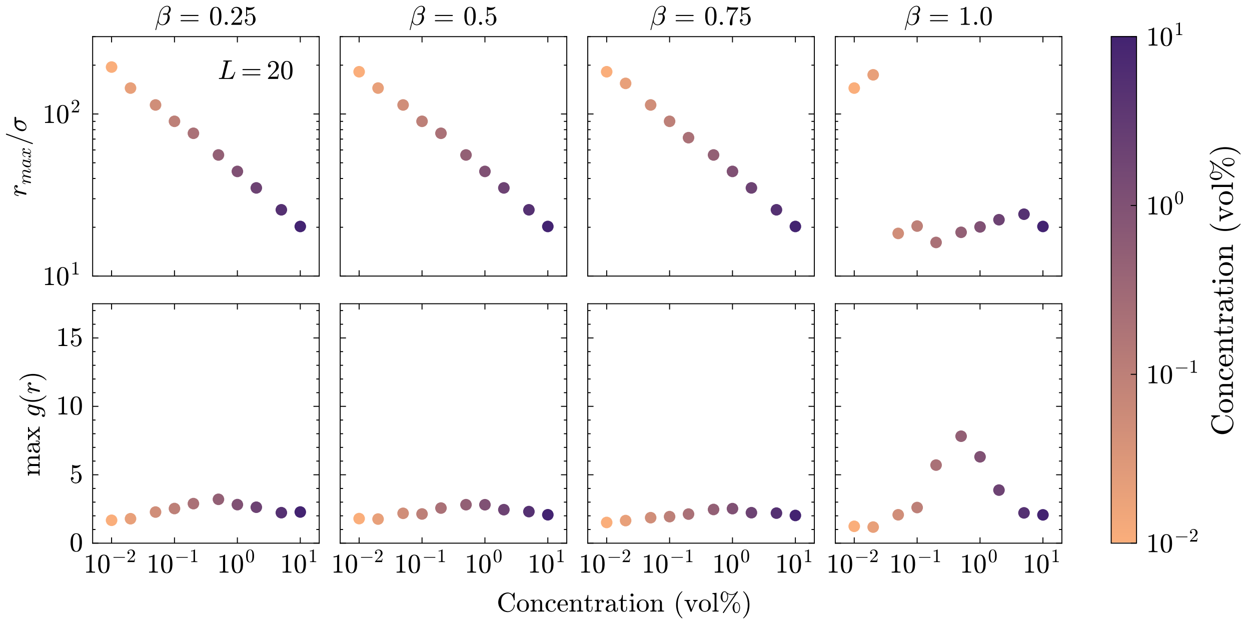

Figure S9: **Dependence of the position and magnitude of the first peak in the radial distribution function $g(r)$ on concentration for various values of β at $L = 20$.** The top row shows the location of the maximum in $g(r)$ as a function of concentration. The bottom row shows the corresponding maximum value of $g(r)$. Colours indicate concentration on a logarithmic scale.

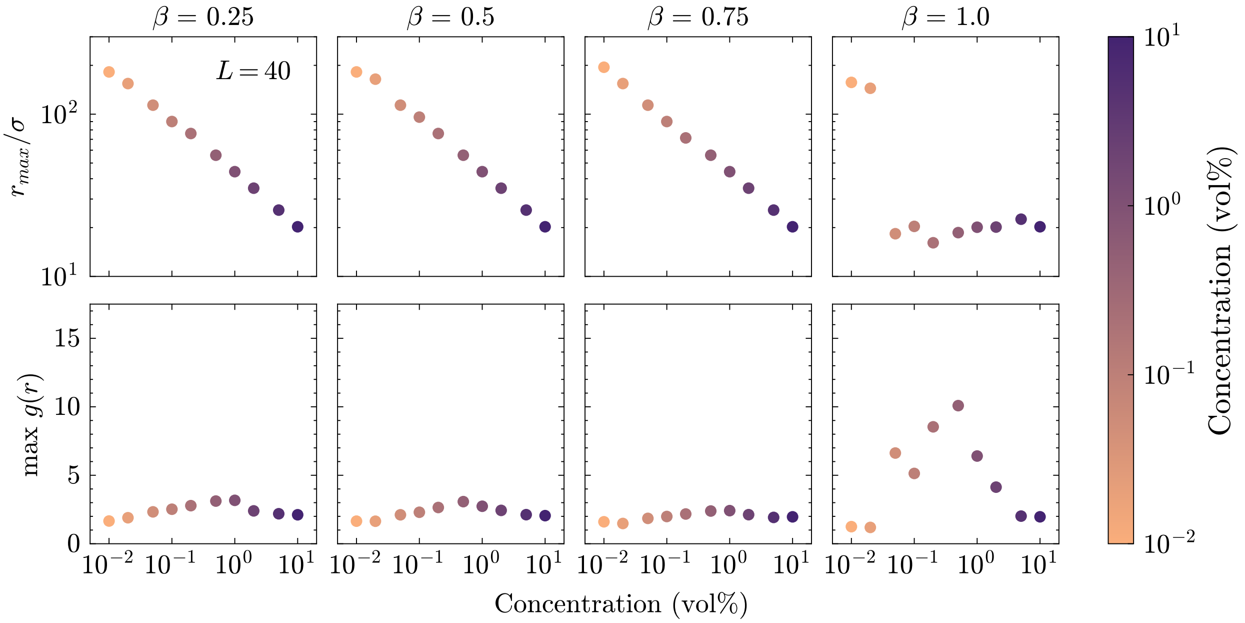

Figure S10: **Dependence of the position and magnitude of the first peak in the radial distribution function $g(r)$ on concentration for various values of β at $L = 40$.** The top row shows the location of the maximum in $g(r)$ as a function of concentration. The bottom row shows the corresponding maximum value of $g(r)$. Colours indicate concentration on a logarithmic scale.

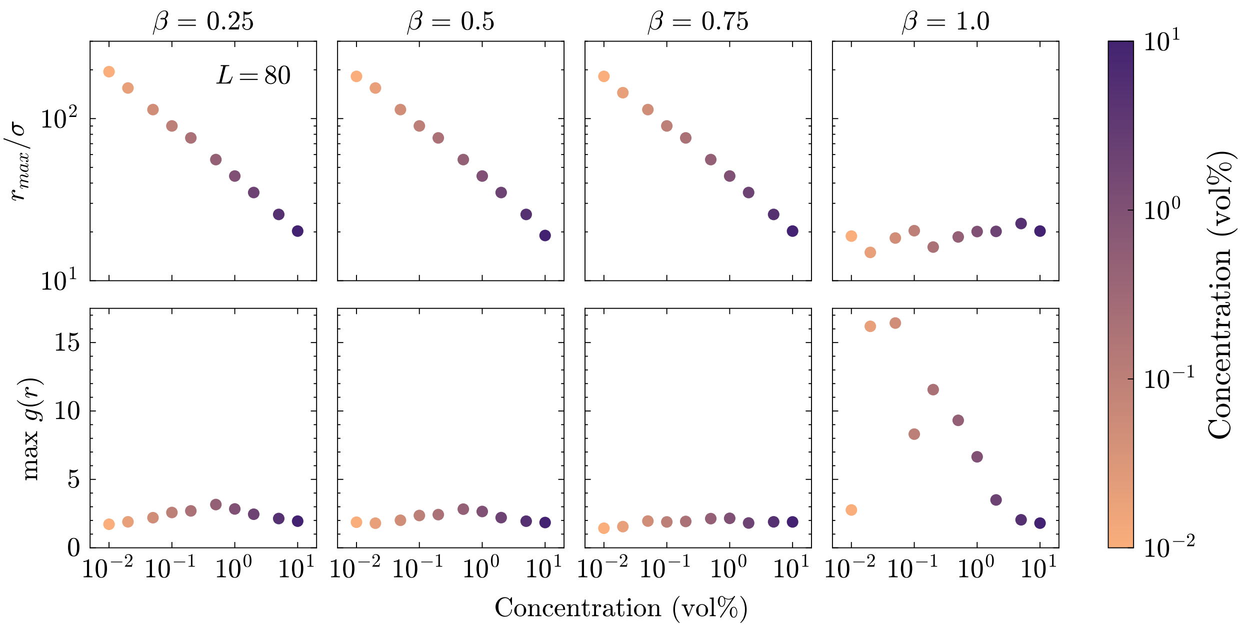

Figure S11: **Dependence of the position and magnitude of the first peak in the radial distribution function $g(r)$ on concentration for various values of β at $L = 80$.** The top row shows the location of the maximum in $g(r)$ as a function of concentration. The bottom row shows the corresponding maximum value of $g(r)$. Colours indicate concentration on a logarithmic scale.

Mean squared displacement of star polyelectrolyte cores

Fig. S12 shows the mean squared displacements of the SPE cores. Despite the structural phase reentrance observed in the system, the results show that the SPEs do not exhibit any dynamical phase reentrance.

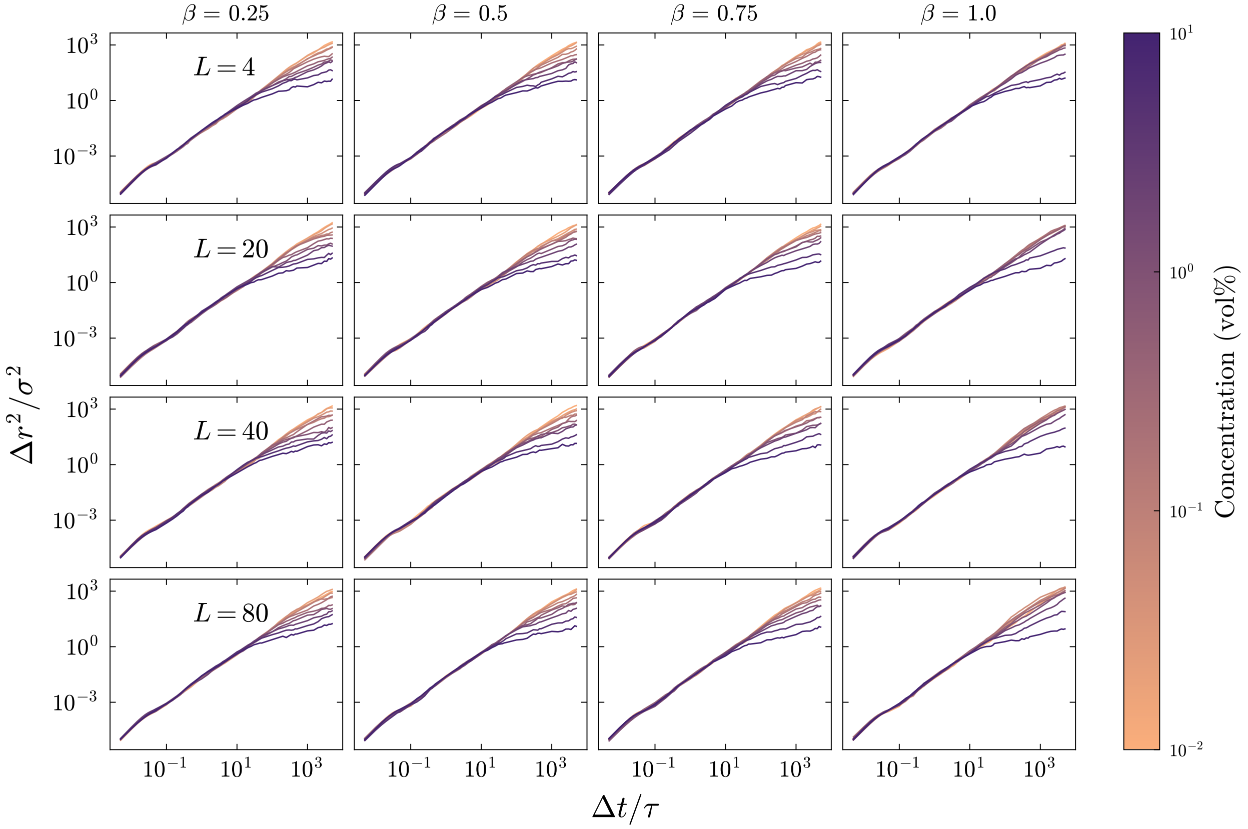

Figure S12: **Mean squared displacement of star polyelectrolyte cores.** Each row shows systems at the fixed LPE length for all concentrations. Columns are at fixed charge ratio.
